# Supplementary figures and images for: Loss of Jag1 cooperates with oncogenic Kras to induce pancreatic cystic neoplasms
Source: Life Sci Alliance. 2020 Dec 2;4(2):e201900503. doi: 10.26508/lsa.201900503 (PMC7756968; doi:10.26508/lsa.201900503)

Jag1

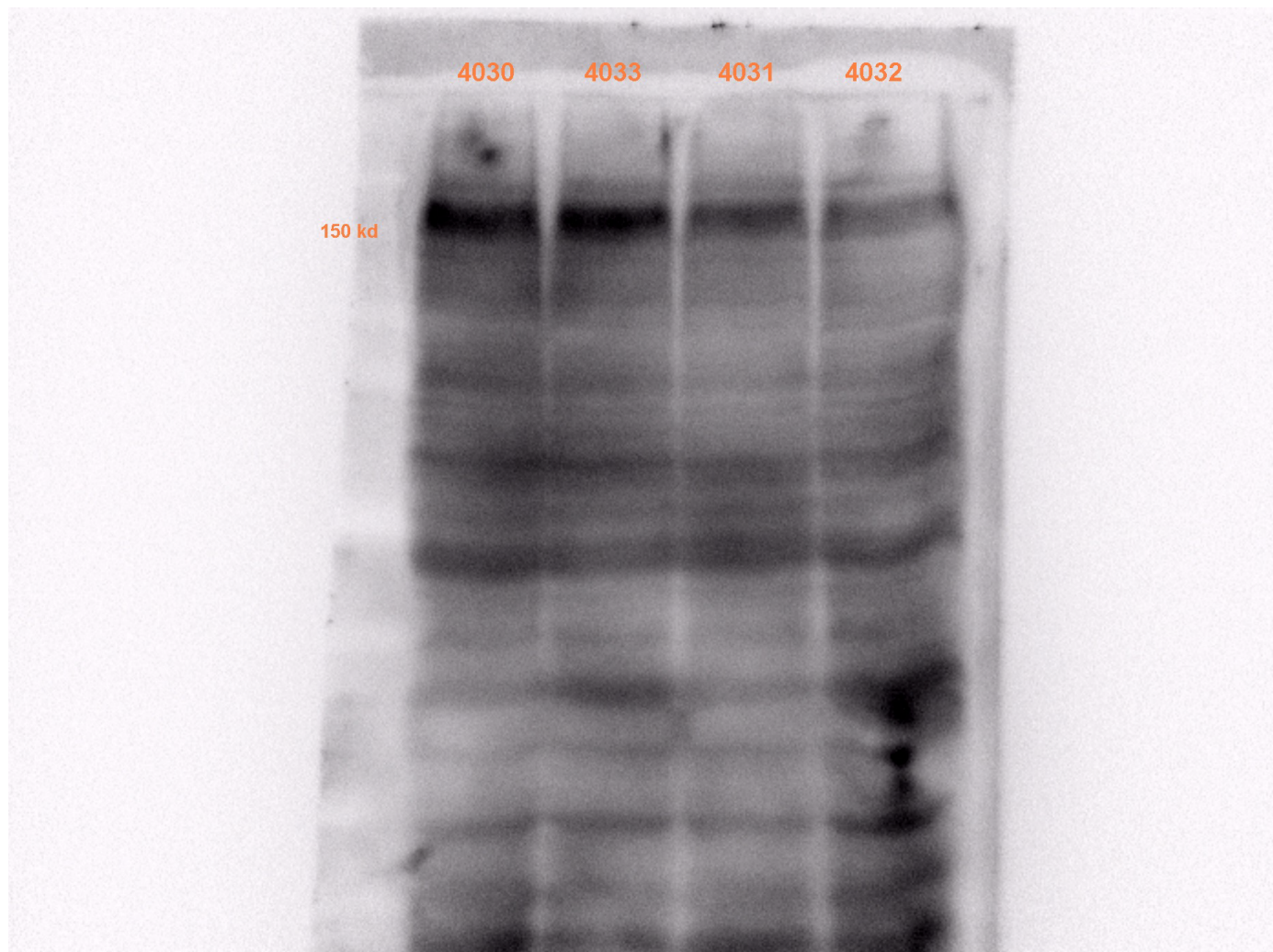

$\beta$ -actin

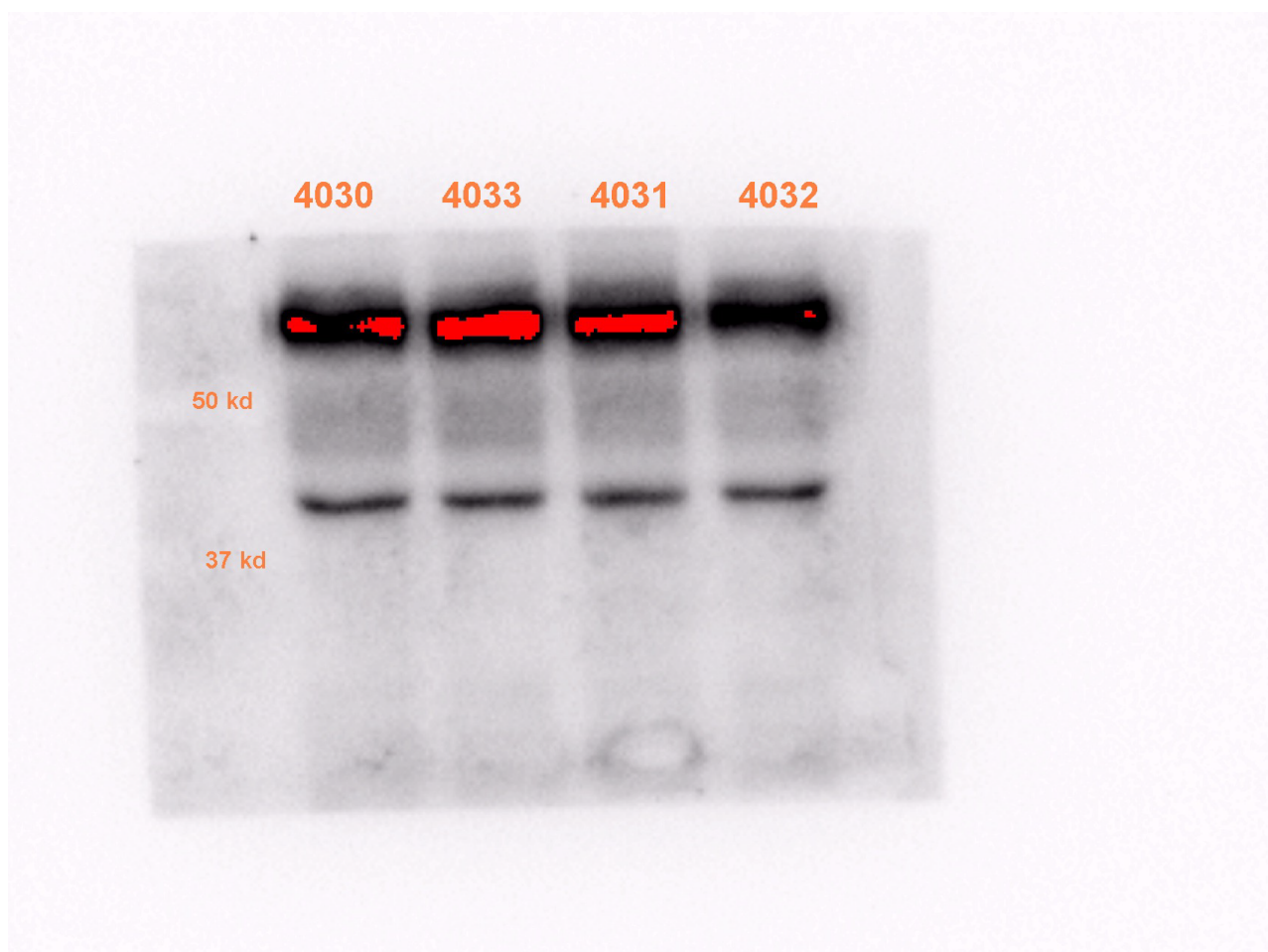

Supplement: Supplementary file 1 [file LSA-2019-00503_SdataF1.pdf]

Sox9

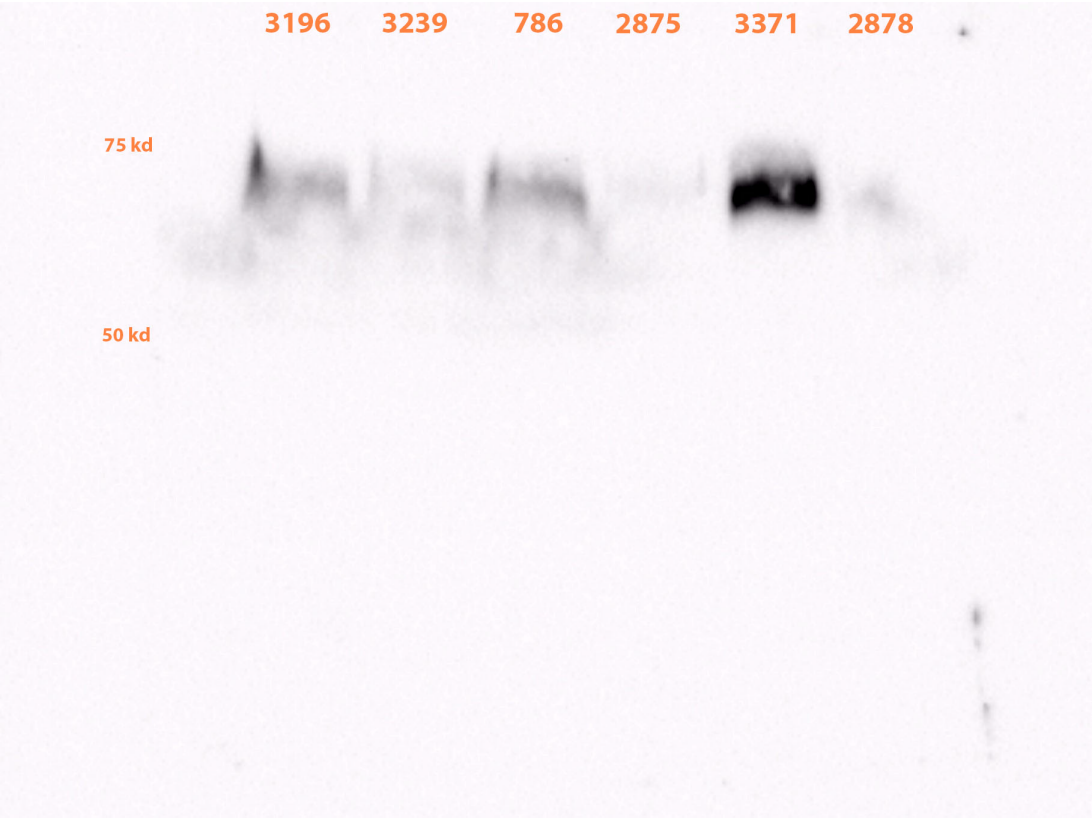

$\beta$ -actin

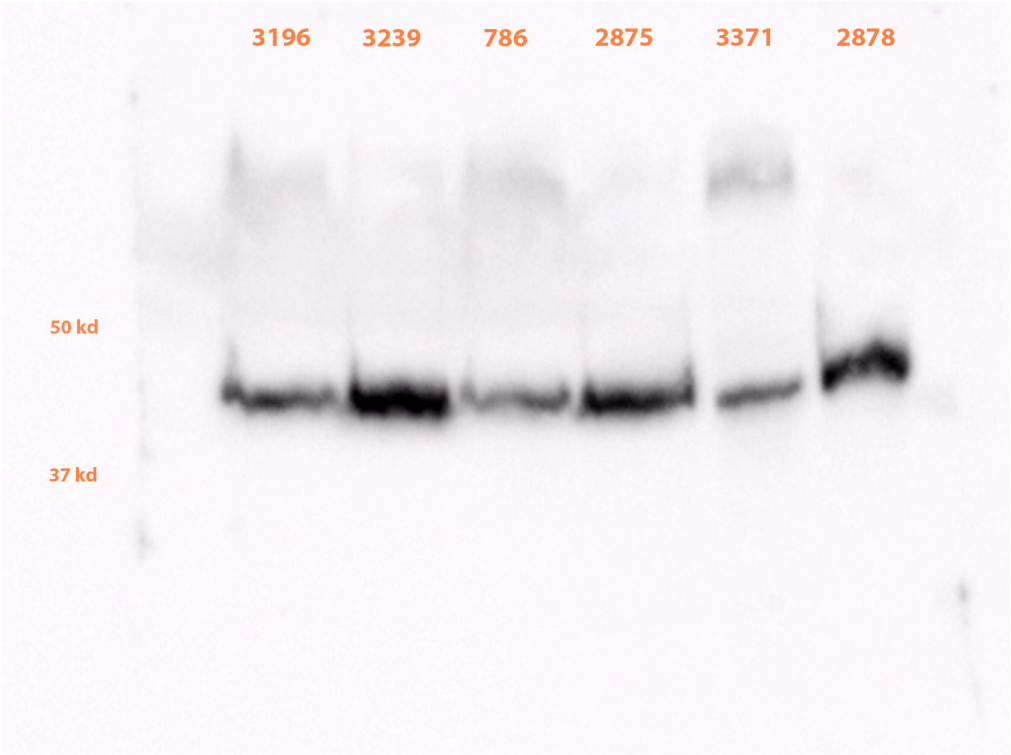

Supplement: Supplementary file 2 [file LSA-2019-00503_SdataF5.pdf]
